# The Effect of Educational Intervention based on BASNEF Model on Decreasing the Cesarean Section Rate among Pregnant Women in Khomain Country

**Published:** 2015-09

**Authors:** Zohreh Arefi, Davod Hekamatpou, Mohammad ali Orouji, Zahra Shaahmadi, Giti Khushemehri, Faramarz Shaahmadi

**Affiliations:** 1 School of Public Health, Kurdistan University of Medical Sciences, Sanandaj, Iran; 2 Faculty of Nursing and Midwifery, Arak University of Medical Sciences, Arak, Iran; 3 School of Public Health, Tehran University of Medical Sciences, Tehran, Iran; 4 Clinical Research Development Center, Imam Khomeini Hospital, Kermanshah University of Medical Sciences, Kermanshah, Iran; 5 Savojbolagh Health Center, Alborz University of Medical Sciences, Karaj, Iran

**Keywords:** Educational intervention, BASNEF model, Cesarean section

## Abstract

**Objective:** Over the past two decades, the incidence of cesarean section in most countries has increased. Cesarean section increases the risk of death and complications in the mother and fetus. Educational interventions based on behavior change models can play an important role in reduce the rate of cesarean section. The aim of our study is investigation the effect of educational intervention based on BASNEF Model on decreasing of cesarean section rate among pregnant women in Khomain County, from June to November 2013.

**Materials and methods:** In this interventional study, 140 nulliparous women who were in their last trimester of pregnancy were randomly allocated to case and control groups. Data gathering instrument was a questionnaire based on BASNEF framework. Data were analyzed by SPSS 14 software.

**Results:** The scores of knowledge, attitudes, subjective norms, and enabling factors in the intervention group than the control group showed a significant difference (p < 0.001). After the study, it was found that 18 women (25.7%) in case group and 42 women (60%) in the control group underwent cesarean section. By Chi-square test showed that the difference in the type of delivery between the two groups was statistically significant (p < 0.001).

**Conclusion:** Design and implement curriculum based on BASNEF can be effective in reducing elective cesarean section.

## Introduction

Cesarean section can be considered as an important and life-saving surgery in the absence of vaginal delivery (1).The most common indications for cesarean section are breech presentation, dystocia, fetal distress and previous cesarean delivery (2). However, in many cases, cesarean section is done without these medical indications that can increase maternal complications, such as infection, bleeding, uterine lacerations, thromboembolic disease, anesthetic complications, and etc. (3-4).

Although mortality and morbidity in cesarean section is higher in comparison with vaginal delivery, this surgery is increasing in the world (5). There is not yet consensus on the ideal for cesarean section rate, but the World Health Organization announced that acceptable rate of cesarean section in the entire world was 10 to 15% in 1985 (6-7). 

The cesarean rate has been increased from 20.5 to 32.8 percent between years of 1996 and 2009 in the US (8). Also during these years, the rate of cesarean section has been raised from 16.3 to 24.8 percent in England (9). 

The prevalence range of cesarean section in Iran is 26 to 60 percent that have been reported in many studies (10-12). According to the World Health Organization report in 2010, the rate of cesarean section in Iran was 41.9 percent (13). 

Attitude, behavior and believe are the factors that determine the delivery in many cases (14). Furthermore, qualitative studies conducted in Iran showed that attitude, subjective norms and perceived behavioral control were influencing factors for choosing cesarean section among pregnant women who were in their third trimester (15-16). 

The need of intervention for reducing cesarean section rate in Iran is essential, because cesarean section rate should reach the goal of the World Health Organization. It is worth mentioning that effective interventions in reducing the cesarean section depend on the factors affecting the mother's decision. There are several health educational models that can be used for seeking causes, analyzing and interpreting healthy behaviors, one of these useful models of behavior change that consider the subjective norms and enabling factors in addition to the effect of knowledge and attitudes has known BASNEF model. This model has various constructs such as attitude, subjective norms (social pressures), enabling factors and behavior intention. This model is used to study and plan for behavior change and to determine the factors that affect the individuals' decision for behavior (17). Efficiency of BASNEF model has been proven in various studies (18-20). The aim of our study is investigation the effect of educational intervention based on BASNEF Model on decreasing of cesarean section rate among pregnant women in Khomain County.

## Materials and methods

This study was approved by the Ethics Committee of Arak University of Medical Sciences (No: 89-100-4). This quasi-experimental study was conducted among nulliparous women who were in their last trimester of pregnancy in Khomain County. Inclusion criteria included women under 35 years old experiencing their first pregnancy that complete the consent form to participate in the study. Total number of sample was 140 women that divided into two groups with 70 women. Questionnaire has been applied for collecting data, and it included the demographic, knowledge, attitude, subjective norms and enabling factor questions. Demographic questions were about her/her husband age, educational level, occupation and income. The number of knowledge, attitude and enabling factor questions were 10, 16 and 7, respectively. Subjective norms were the individuals who have impact on woman decision for the type of delivery such as her husband, mother, mother in-law, friends, health personnel and physician. 

The validity of the questionnaire was determined by six faculty members, obstetricians and gynecologists and its reliability was confirmed using test-retest method (α = 0.90).

Data analysis was done in SPSS 14 using paired t-test, independent t-test, chi-square test, McNemar's test and descriptive statistics.

## Results

The mean and standard deviation of age in case and control groups was 25.32 ± 3.36 and 26.12 ± 3.24 respectively. Also other demographic variables have shown in table 1. There were not statistically significant differences between demographic variables in the two groups (p > 0.05) (Table 1).

There was a significant difference between knowledge’s score before and after the intervention in the case group (p < 0.001), but no significant differences were observed in the knowledge’s score of control group (p = 0.918). Although Attitude’s scores between before and after the intervention in the case group have shown significant differences (p < 0.001), there was no significant difference between Attitude’s scores in the control group (p = 0.562). However, it was shown that the enabling factors in the case group have statistical difference (p < 0.001), but this difference was not statistically significant in the control group (p = 0.281) (Table 2).

**Table 1:** Demographic characteristic of pregnant women in case and control groups

		Case	Control	P value
Age (Year) (mean ± SD)		25.32 ± 3.36	26.12 ± 3.24	0. 031
Mother’s job [n(%)]	Housekeeper	64 (91.4%)	63 (90%)	0.347
	employed	6 (8.6%)	7 (10%)	
Spouse’s job [n(%)]	Unemployed	7 (10%)	4 (5.7%)	0.382
	Employed	63 (90%)	66 (94.3%)	
Mother's education (High school and Upper) [n(%)]		29 (41.4%)	27 (38.6%)	0.681
Spouse’s Education (High school and Upper) [n(%)]		22 (31.3%)	19 (27.1%)	0.602

**Table 2:** Mean and standard deviation scores of knowledge, attitude and enabling factors among pregnant women in case and control groups

		**Before the intervention** **Mean ± SD**	**After the intervention** **Mean ± SD**	**P value**
Awareness	Case	43.5 ± 9.2	82.5 ± 10	0.001
	Control	42.1 ± 11	45 ± 15.7	0.918
Attitude	Case	55.5 ± 12.2	66.5 ± 11.1	0.001
	Control	56.4 ± 13.1	52.1 ± 15.9	0.562
Enabling factors	Case	56.2 ± 21	77 ± 22.5	< 0.001
	Control	59.5 ± 20.6	57.5 ± 22.6	0.281

**Table 3:** Distribution of Subjective norms among pregnant women in case and control groups

	**Spouse**		**Mother**		**Mother-in-law**		**Friends**		**Health ** **personnel**		**Physician**	
	**Case**	**Control**	**Case**	**Control**	**Case**	**Control**	**Case**	**Control**	**Case**	**Control**	**Case**	**Control**
Before (Frequency)	79	86	81	92	30	34	27	47	72	75	60	54
After (Frequency)	82	80	83	85	35	36	52	45	97	79	85	54
P Value	0.857	0.256	0.477	0.06	0.499	0.476	< 0.001	0.313	< 0.001	0.291	< 0.001	0.321

By comparison subjective norm’s scores with Mc Nemar's test before and after intervention in the case group had shown that individuals such as friends, health personnel and physician affect mother decision (Table 3).

At the end of the study 52 women (74.3%) in case group performed vaginal delivery and 18 women (25.7%) had caesarean section. While in the control group, 28 women (40%) underwent elective vaginal delivery and 42 women (60%) had caesarean section. The chi-square test showed that the difference in the type of delivery between the two groups was statistically significant (p < 0.001).

## Discussion

The mean knowledge score of pregnant women about the benefits of vaginal delivery in the two groups after the intervention has been raised and the difference was statistically significant.

Also in other studies, educational interventions have increased the awareness of pregnant women about the benefits of vaginal delivery and complications of cesarean section (21- 22). Tavasoli concluded in his study that the knowledge of pregnant women should be increased to reduce the incidence of cesarean section and its complications in mothers and infants (23).

There are some related studies in this subject such as Charkazi’s study about the effect of maternal awareness on their improved breastfeeding behavior (24) and the Moez’s study about women's awareness increase and the use of safe contraceptive methods (25).

After the intervention, the attitude’s scores of pregnant women in the case group meaningfully have been increased. Women's attitudes affect the choice of delivery method. At the study of Waldenström and etc., women who still had negative attitude toward vaginal delivery have chosen cesarean section 3 to 6 times higher than vaginal delivery as the selected method (26).

Subjective norms (individuals) such as friends, health personnel and physician affect mother decision about the type of delivery in this study. Educating mothers alone has insignificant role in choosing the appropriate health behavior. An extensive training program will be needed to change the subjective norms that include women relatives such as doctors, health personnel and friends.

Although the behavior is a multifactorial phenomenon, the programs that publish health information without considering the enabling factors will fail to change behavior.

In this study, the use of educational resources about the benefits of vaginal delivery and complications of cesarean section, organizing training classes, and providing home study educational materials have been considered as enabling factors.

The mean score of enabling factors in the case group has been significantly increased after intervention. Also Moeini and etc. investigated the predictive power of the enabling factors associated with increased physical activity in their study (27), and enabling factors of women's self-care behaviors have been studied in Izadirad research (28).

## Conclusion

In our study, some factors have a positive impact on the pregnant women performance such as knowledge, attitudes, enabling and subjective norms. Thus, planning the intervention on the basis of the BASNEF model can be a significant factor to prevent the increase in cesarean section’s rate.
